# Medication adherence in a German cohort of patients with advanced or metastatic breast cancer: results of the BRE-BY-MED study

**DOI:** 10.1186/s12885-026-15641-y

**Published:** 2026-01-30

**Authors:** Lilly Sophia Brandstetter, Anna Grau, Max Müller-Reiter, Jessica Salmen, Jacqueline Müller-Nordhorn, Nikola Beck, Peter Heuschmann, Stefan Störk, Achim Wöckel, Jens-Peter Reese

**Affiliations:** 1 Institute for Clinical Epidemiology and Biometry, Julius-Maximilian University Würzburg, Am Schwarzenberg 15, 97078 Würzburg, Germany; 2 Department of Gynaecology and Obstetrics, University Hospital of Würzburg, Würzburg, Germany; 3 Bavarian Cancer Registry, Bavarian Health and Food Safety Authority, Erlangen, Germany; 4 Institute of Medical Data Science, University Hospital Würzburg, Würzburg, Germany; 5 Department of Clinical Research & Epidemiology, Comprehensive Heart Failure Center, University Hospital Würzburg, Würzburg, Germany; 6 Department of Internal Medicine I, University Hospital Würzburg, Würzburg, Germany; 7 Faculty of Health Sciences, Technische Hochschule Mittelhessen, University of Applied Sciences, Giessen, Germany

**Keywords:** Breast cancer, Metastatic breast cancer, Adherence, Persistence, Routine data, Cancer registry data, Patient reported outcomes

## Abstract

**Background:**

In patients with advanced or metastatic breast cancer (mBC), adherence to treatment, supportive therapy, and comorbidity medication is important for prolonging survival time, reducing symptom burden and side effects, and improving quality of life. The aim of the present study was to determine medication adherence and potential influencing factors in patients with advanced or mBC.

**Methods:**

Adults with advanced or mBC treated at the University Hospital Würzburg living in Bavaria, were included in the BRE-BY-MED “Breast Cancer Care in Bavaria for Patients with Metastatic Disease” cohort study (DRKS00026601). Self-reported adherence was analysed using data from patients at baseline, and after 3-, 6-, and 12-months of follow-up, and using Bavarian cancer registry data.

**Results:**

Between July 2022 and February 2024, 93 patients (median age 57 years; IQR = 48–64), were consecutively enrolled in the BRE-BY-MED study. At baseline, 75.3% (*n* = 70) and 86.0% (*n* = 80) of the patients reported being adherent and dosage adherent, respectively. During the follow-up period, no relevant changes in adherence levels were observed. Supportive therapy and medication for comorbidities were most affected by non-adherence. The most common reasons for non-adherence were medication beliefs, forgetfulness, and side effects. For 81.2% (*n* = 69) of the patients, complementary cancer registry data were available and complete, and therapy persistence was found in 78.3% (*n* = 54) of these patients.

**Conclusion:**

The observed adherence rates indicate that tailored interventions to improve adherence to supportive therapy and medication for comorbidities in patients with advanced or mBC are needed, particularly considering the reasons for non-adherence.

## Introduction

Advanced or metastatic breast cancer (advanced or mBC) occurs in approximately 30% of cases and is defined as breast cancer (BC) that has metastasised to other parts of the body [1–4]. The median survival time for patients is 2 to 4 years [1–3]. Adherence to mBC and supportive therapy is of paramount importance in prolonging patients’ survival time, as well as reducing the symptom burden and potential side effects, thereby improving quality of life (QoL) [1, 5]. Furthermore, the prevalence of at least one comorbidity among patients with different cancer types has been documented to be approximately 70% [6]. This additional burden of disease is accompanied by the challenge of managing the pharmacological treatment for these comorbidities while undergoing cancer treatment [7]. Moreover, it has been shown that adherence to comorbidity medication in cancer patients was associated with better health-related QoL [7].

**Fig. 1:**
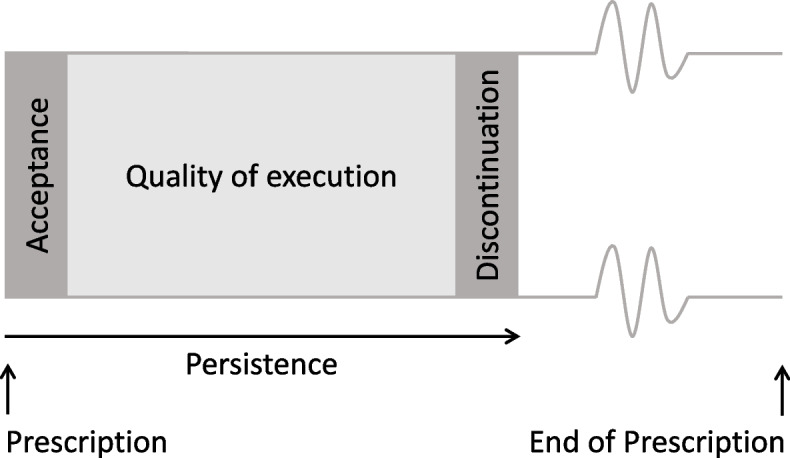
Adherence phenomena (after Vrijens and Urquhart, 2005 [9])

Adherence, is defined as “the extent to which a person’s behaviour – taking medication, following a diet, and/or executing lifestyle changes – corresponds with agreed recommendations from a health care provider” [8]. Non-adherence can have a number of consequences, including reduced therapeutic efficiency and subsequent higher morbidity and mortality, as well as economic consequences for the healthcare system, such as avoidable hospitalisations, additional healthcare visits, or costs created by unused drugs [9]. The concept of adherence encompasses two different phenomena: persistence and quality of execution (Fig. 1). The term ‘persistence’ is defined as the duration for which a patient adheres to the prescription, while ‘quality of execution’ is defined as the manner in which a patient adheres to the prescription, in terms of regimen or other instructions in case of non-pharmacological interventions [10]. Adherence can be measured by various methods: (1) subjective methods such as self-report in the form of interviews or questionnaires, and (2) objective methods, which include the measurement of biomarkers, pill counting, electronic monitoring, or the analysis of routine data [11, 12]. There is no reference standard for adherence measurement [11]. It is recommended to apply more than one method to counteract the limitations of each individual method. Self-report adherence is highly susceptible to manipulation, reporting bias due to social desirability, and recall bias [11, 12]. For routine data, the assumption is made that the medication is taken as prescribed [13].

Adherence to drug therapy is influenced by various factors including, but not limited to, disease severity and symptom burden, side effects, polypharmacy, comorbidities, patients’ attitude towards their disease, psychological challenges, patients’ cognitive state, physician–patient relationship, and several sociodemographic factors [14–31]. Particularly, cancer medication is often characterised by complex dosing regimens and a high potential for drug-drug and drug-food interactions [32–34]. Furthermore, in BC, an advanced disease has been identified as risk factor for non-adherence [1, 27, 35, 36].

Recent reviews have focused on patients with early-stage BC and adherence to BC therapies [14–31, 33, 37]. The reported adherence to different oral cancer drugs varied between 12 and 98%, depending on the type of BC therapy and the type of adherence measurement [14, 15, 17–20, 31, 37]. Very few studies focused on patients with advanced or mBC and reported adherence rates between 65 and 96% [1, 38, 39]. These studies relied on pill counting, or surveys of patients, caregiver, or physicians. Identified factors influencing mBC patients’ adherence were the (perceived) treatment effectiveness, side effects and dosing regimen [1, 38]. None of these studies assessed adherence to supportive therapies or comorbidity medication. Yet, as described above, the combination of adherence to cancer medication, to the supportive therapy, and to medication for comorbidities in advanced or mBC patients is highly important. Therefore, the aim of the present study was to determine medication adherence in a German cohort of patients with advanced mBC using self-reported information and linked data from the Bavarian cancer registry.

## Methods

### Study design

The data was obtained from subjects participating in the BRE-BY-MED “Breast Cancer Care in Bavaria for Patients with Metastatic Disease” study (DRKS00026601). BRE-BY-MED was a prospective cohort study conducted at the Department of Gynaecology and Obstetrics of the University Hospital Würzburg. The primary objective of the study was to estimate the prevalence of guideline concordant treatment [40].

### Inclusion and exclusion criteria

The BRE-BY-MED study comprised adult patients of both sexes diagnosed with prevalent or incident advanced or mBC residing in Bavaria and who provided written informed consent. Within the study, advanced or mBC was defined by ICD-10 Code C50 or C76-C80 and TNM classification pTx-pNx-M1 or pTx-pN(1–3)-Mx. The exclusion criteria comprised patients < 18 years, those with non-advanced or non-mBC and those not residing in Bavaria. This was done in order to ensure a most representative study population of clinical routine care.

### Data collection

**Fig. 2:**
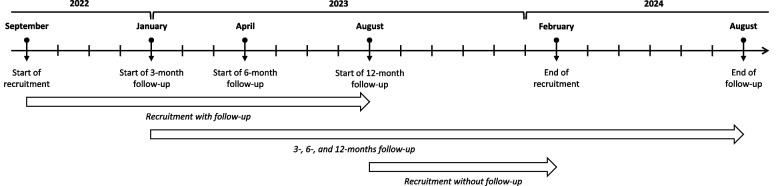
Timeline of recruitment in the BRE-BY-MED study

In July 2022, a pilot study was conducted to ascertain the feasibility of the design of the BRE-BY-MED study. Data were collected at baseline (study inclusion) and after a 1-month follow-up period. Patients were recruited in the main study between September 2022 and February 2024. Advanced mBC patients were informed by the BRE-BY-MED study staff and invited to participate. Following the provision of written informed consent, patients completed a baseline survey comprising information on sociodemographic factors (i.e. age, sex, living situation, support by relatives, scholar education, smoking status, body height and weight, and several patient reported outcomes (PROs; as described below), and medication adherence. Patients recruited between October 2022 and August 2023 were followed for 12 months: these patients received mailed questionnaires on medication adherence at 3, 6, and 12 months after study inclusion. Patients recruited between August 2023 and February 2024 only completed the baseline survey without follow-up. Figure 2 depicts the timeline of recruitment in the BRE-BY-MED study.

### Adherence measurement

#### Self-reported adherence

Adherence in terms of persistence and quality of execution was measured using the medication adherence questionnaire that was developed and validated within the SANO study (the structured ambulatory post-stroke care program for outpatient aftercare in patients with ischaemic stroke in Germany) [41]. Patients were asked about the affected medication (i.e. mBC treatment, supportive therapy, comorbidity medication), the underlying causes of non-adherence (i.e. forgetfulness, medication beliefs, side effects, or frequent medication changes), and on their comprehension of their medication. For the affected medication and the underlying causes of non-adherence multiple answers were possible. The adherence is reported as proportion of adherent patients.

#### Cancer registry data

In Germany, the Cancer Registry Act, which came into force in 2013, requires standardised collection and reporting of cancer data in all federal states, with the objective of ensuring consistency and quality in cancer surveillance. Germany's cancer registry system is a comprehensive network of clinical-epidemiological registries [42].

For the present study, we used data from the Bavarian cancer registry to analyse adherence in terms of persistence. These data included information on systemic therapies starting from initial diagnosis of metastasis to the time of the 12-month follow-up. The routine data were queried for individual patients using the following identifying variables: name, surname, date of birth, place of residence, and postal code. Data were transferred via a data protection-compliant cloud and imported into the central database. The persistence is reported proportion of patients without therapy pauses or discontinuation.

### Patient reported outcomes

The perceived health status and QoL were assessed using the EORTC-QL-2 (questions 29 and 30 of the EORTC-QLQ-C30, version 3 [43, 44]). The assessment of depressive and anxiety symptoms was conducted using the PHQ-4 [45]. Physical functioning was assessed using the PROMIS® v2.0 Physical Function Short Form 4a (PROMIS-PF-4a) and reported as T-score [46, 47]. The current pain level of the patients was measured using an 11-point Likert scale, ranging from 0 = “no pain” to 10 = “strongest pain”.

The assessment of treatment-related side effects included infections, metabolic and nutritional disorders, cardiovascular side effects, side effects of the kidney or urinary tract, the respiratory system, the eyes, the nervous system, the gastro-intestinal system, the skin, and the musculo-skeletal system. The inclusion of these side effects was determined based on expert opinion (study physicians). The impairment caused by the side effects experienced by the patients was measured on a 5-point Likert scale ranging from 1 = “no impairment” to 5 = “severe impairment”. For the present analysis, the impairment was dichotomised by at least slight impairment (= 2 points). The detailed description of the assessment of PROs in the BRE-BY-MED study was published elsewhere [48].

Additionally assessed PROs were the CARE Scale, measuring ten different aspects of satisfaction with physician’s empathy on a 5-point Likert scale, ranging from 1 = full agreement to 5 = disagreement; and 6 = not assessable [49]. The summary score for all ten items ranges from 10 to 50, with lower scores representing higher satisfaction with physician’s empathy. Information needs were assessed for eight different aspects of BC therapy and measured from feeling not informed to feeling fully informed. For each aspect, patients could further state whether they wished for more information. Support needs were assessed for six health care areas and measured on a 5-point Likert scale, ranging from 1 = no need for support to 5 = high need for support.

### Clinical data

The treating physicians entered information on diagnosis (ICD-10 code), type of systemic BC therapy, the Charlson Comorbidity Index (CCI), menopausal status, and HR/HER2 status into an electronic case report form (eCRF).

### Statistical analyses

Descriptive statistics are reported as count (percent), or median (interquartile range [IQR]), as appropriate. Univariate subgroup analyses for baseline medication adherence were performed for sociodemographics, clinical characteristics, and PROs. For the present analysis, the time since first diagnosis of metastasis, number of concurrent metastases, perceived health status, QoL, physical functioning, pain level, and physician’s empathy were each classified into two groups using the median split.

## Results

### Participants

**Fig. 3:**
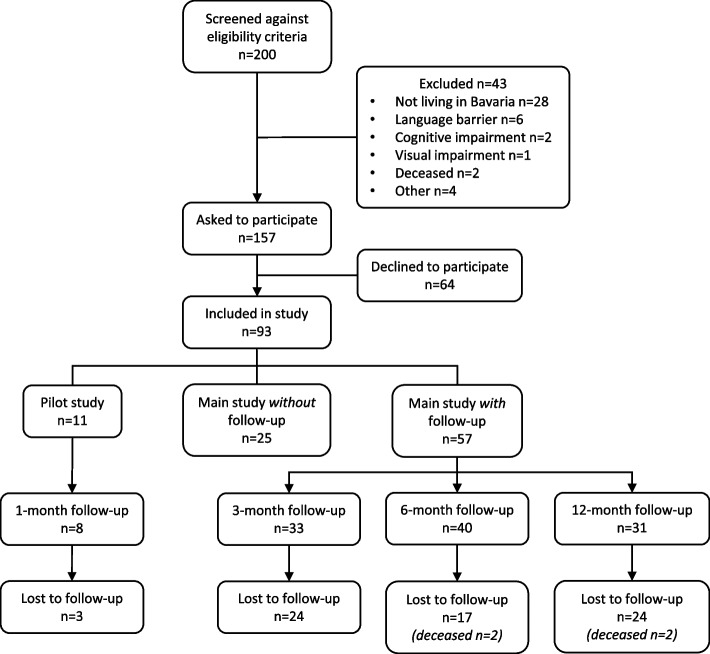
Flow-chart of the BRE-BY-MED study

93 patients were consecutively enrolled in the BRE-BY-MED study, of which 11 were included in the pilot study and 82 in the main study (Fig. 3). In the main study, 57 patients were recruited between September 2022 and August 2023 and were thereby followed-up for 12 months. In the pilot study, eight patients provided information at 1-month follow-up (response rate 72.2%). In the main study, 33, 40 and 31 patients provided information at 3-, 6-, and 12-months follow-up, respectively (response rates: 57.9%, 70.2%, 56.4%). 25 patients were recruited between September 2023 and February 2024 (without follow-up).

The median age was 57 years (IQR = 48–64), one patient was male. Types of BC therapy were 48.4% (*n* = 45) chemotherapy, 59.1% (*n* = 55) endocrine therapy, 34.4% (*n* = 32) targeted therapy, 53.8% (*n* = 50) osteoprotective therapy, and 3.2% (*n* = 3) immunotherapy. Patients received BC therapy in oral or intravenous forms, or in combination of both. Of note, adherence to BC therapy was only assessed in the oral modality.

Concerning the additional PROs, the median satisfaction with physician's empathy was good with a median of 14.0 (IQR 10.8–24.0) points on the CARE scale (*missing n* = *11*). 45.2% (*n* = 42) of the patients reported in at least one category that they felt slight or not informed (*missing n* = *6*). 38.7% (n = 36) of the patients reported moderate to high support needs in at least one category (*missing n* = *5*). The detailed description of the study population was published elsewhere [48].

### Medication adherence

#### Self-reported adherence

At baseline 75.3% (*n* = 70) and 86.0% (*n* = 80) of the patients reported to be adherent in terms of persistence and quality of execution, respectively (Table 1). No difference in persistence during the follow-up period was observed. The quality of execution slightly worsened over time. In general, supportive therapy and medication for comorbidities were most affected by non-adherence. In Table 2 the affected medications or combinations of affected medications are depicted, respectively. The detailed description of the specific medication was published elsewhere [48].

**Table 1:** Medication adherence at baseline (*n *= 93), 1-/3- (*n* = 41), 6- (*n* = 40), and 12-month (*n* = 31) follow-up

	*Baseline (n = 93)*	*1-/3-month follow-up (n = 41)*	*6-month follow-up (n = 40)*	*12-month follow-up (n = 31)*
*Non-Adherence in terms of persistence (n, %)*	23 (24.7)	15 (36.6)	12 (30.0)	10 (32.3)
	*(missing n* = *6)*			
Affected medication (n, %)*				
BC therapy	3 (13.0)	4 (26.7)	4 (33.3)	1 (10.0)
Supportive therapy	20 (87.0)	5 (33.3)	7 (58.3)	9 (90.0)
Medication for comorbidities	6 (26.1)	8 (53.3)	5 (41.7)	1 (10.0)
* Frequency of medication intake (n, %)*				
More often than prescribed	1 (4.3)	0 (0)	2 (16.7)	0 (0)
Sometimes more often, sometimes less often than prescribed	13 (56.5)	7 (46.7)	4 (33.3)	4 (40.0)
Less often than prescribed	9 (39.1)	6 (40.0)	5 (41.7)	6 (60.0)
		*(missing n* = *2)*	*(missing n* = *1)*	
Reasons (n, %)*				
Forgetfulness	5 (21.7)	2 (13.3)	2 (16.7)	1 (10.0)
Side effects	2 (8.7)	4 (26.7)	1 (8.3)	3 (30.0)
Medication beliefs	17 (73.9)	6 (46.7)	5 (41.6)	8 (80.0)
Medication changes/drug burden	0 (0)	0 (0)	1 (8.3)	0 (0)
		*(missing n* = *3)*	*(missing n* = *3)*	
*Non-adherence in terms of quality of execution (n, %)*	13 (14.0)	10 (24.4)	8 (20.0)	11 (35.5)
	*(missing n* = *8)*	*(missing n* = *2)*	*(missing n* = *2)*	*(missing n* = *3)*
Affected medication (n, %)*				
BC therapy	1 (7.7)	1 (10.0)	1 (12.5)	1 (9.1)
Supportive therapy	11 (84.6)	5 (50.0)	5 (62.5)	10 (90.9)
Medication for comorbidities	4 (30.8)	5 (50.0)	2 (25.0)	2 (18.2)
* Dosages taken (n, %)*				
Higher dosage than prescribed	0 (0)	0 (0)	0 (0)	1 (9.1)
Sometimes a higher, sometimes a lower dosage than prescribed	5 (38.5)	3 (30.0)	0 (0)	3 (27.3)
Lower dosage than prescribed	8 (61.5)	7 (70.0)	7 (87.5)	7 (63.6)
			*(missing n* = *1)*	
Reasons (n, %)*				
Forgetfulness	1 (7.7)	1 (10.0)	2 (25.0)	1 (9.1)
Side effects	5 (38.5)	3 (30.0)	3 (37.5)	5 (45.5)
Comorbidities	1 (7.7)	1 (10.0)	0 (0)	0 (0)
Drug burden	1 (7.7)	1 (10.0)	0 (0)	0 (0)
Medication beliefs	1 (7.7)	1 (10.0)	1 (12.5)	3 (27.3)
		*(missing n* = *2)*	*(missing n* = *6)*	
*Time point of medication intake (n, %)*				
At certain times of the day	75 (80.6)	35 (85.4)	35 (87.5)	27 (87.1)
When feeling well	1 (1.1)	0 (0)	2 (5.0)	0 (0)
When feeling unwell	3 (3.2)	3 (7.3)	0 (0)	1 (3.2)
When I remember to take them	0 (0)	0 (0)	2 (5.0)	2 (6.4)
	*(missing n* = *14)*	*(missing n* = *3)*	*(missing n* = *1)*	*(missing n* = *1)*
*Knowing why the medication needs to be taken (n, %)*				
Yes	83 (89.2)	38 (92.7)	39 (97.5)	29 (93.5)
Unsure	0 (0)	2 (4.9)	0 (0)	1 (3.2)
No	0 (0)	0 (0)	0 (0)	0 (0)
	*(missing n* = *10)*	*(missing n* = *1)*	*(missing n* = *1)*	*(missing n* = *1)*
*Knowledge about side effects (n, %)*				
Yes	83 (89.2)	38 (92.7)	39 (97.5)	29 (93.5)
Unsure	0 (0)	2 (4.9)	0 (0)	1 (3.2)
No	0 (0)	0 (0)	0 (0)	0 (0)
	*(missing n* = *10)*	*(missing n* = *1)*	*(missing n* = *1)*	*(missing n* = *1)*
Source of information about side effects (n, %)^*^				
Physician	70 (75.4)	31 (75.6)	34 (85.0)	21 (67.7)
Pharmacist	23 (24.7)	10 (24.4)	14 (35.0)	6 (19.4)
Medication package leaflet	47 (50.5)	24 (58.5)	28 (70.0)	25 (80.6)
Internet	17 (18.3)	14 (34.1)	11 (27.5)	14 (45.2)
Other patients	0 (0)	1 (2.4)	0 (0)	0 (0)
Not informed	5 (5.4)	3 (7.3)	1 (2.5)	2 (6.5)

^*﻿^*More than one answer could be selected per patient*

**Table 2:** Separate analysis of the affected medication by non-adherence at baseline

	***Number of non-adherent patients in terms of persistence (n* = *23)***	***Number of non-adherent patients in terms of quality of execution (n* = *13)***
BC therapy only	1	0
BC and supportive therapy	1	1
BC and medication for comorbidities	0	0
Supportive therapy only	15	8
Supportive therapy and medication for comorbidities	3	2
Medication for comorbidities only	2	2
BC therapy, supportive therapy and medication for comorbidities	1	0

Regardless of the time of observation, the most frequently cited reasons for non-adherence in terms of persistence were medication beliefs and forgetfulness, as well as side effects for non-adherence in terms of quality of execution. In addition, most patients took their medications at certain times of the day, knew why they had to take the medication, and knew about the potential side effects. Common sources of information on potential side effects were the treating physician, the pharmacist, and the medication package leaflet.

#### Cancer registry data

**Fig. 4:**
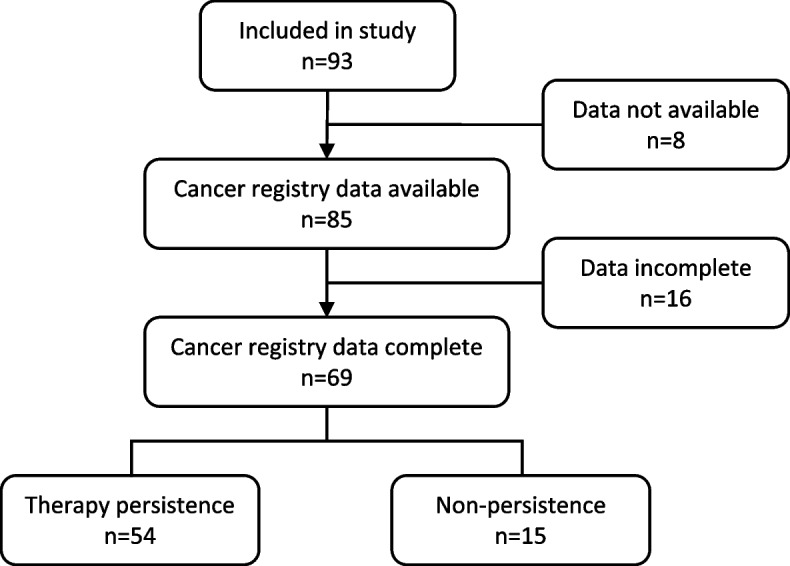
Flow-chart for the Bavarian cancer registry data in the BRE-BY-MED study

For 91.4% (*n* = 85) of the patients, data from the Bavarian cancer registry were available (Fig. 4). For the remaining 8 patients, no treatment data was submitted to the cancer registry. For 81.2% (*n* = 69) of these patients, information on the systemic therapy was complete. For the remaining 16 patients, the end date for a treatment was missing.

In total, therapy persistence was found in 78.3% (*n* = 54) of the patients. In 14.5% (*n* = 10) of the patients, therapy pauses of two and four months (*n* = 6) up to five years (*n* = 4) were detected. For three patients, end of therapy was documented without further entries for therapies. For one patient, it was recorded that therapy was discontinued because the patient refused further therapy.

#### Influencing factors on adherence in patients with advanced or mBC

At baseline, patients who were younger, lived in a partnership, were supported by a relative, were premenopausal, received chemotherapy, had a higher satisfaction with physician’s empathy, and had moderate to high support needs in at least one health care area were more frequently non-adherent than their respective counterparts (Table 3). A significant association was only observed for slight impairment by metabolic and nutritional disorders or by side effects of the nervous system.

**Table 3:** Subgroup analyses for medication adherence in terms of persistence at baseline

	**Adherent (*n* = 64)**^**a**^	**Non-adherent (*n* = 23)**^**a**^	***p* value**
*Age groups*			0.18
18–49 years	18 (28.1)	10 (43.5)	
50–64 years	30 (46.9)	11 (47.8)	
≥ 65 years	16 (25.0)	2 (8.7)	
* Living in a partnership (missing n* = *18)*	36 (56.3)	17 (73.9)	0.36
* Supported by relative (missing n* = *11)*	47 (73.4)	22 (95.7)	0.16
* Grammar school education* *(missing n* = *1)*	20 (31.3)	6 (26.1)	0.64
* Smoker (active/former/passive) (missing n* = *3)*	35 (54.7)	13 (56.5)	0.77
*BMI (missing n* = *8)*			0.31
Underweight	4 (6.3)	0 (0)	
Normal weight	24 (37.5)	7 (30.4)	
Overweight	17 (26.6)	10 (43.5)	
Obese	15 (23.4)	4 (17.4)	
* Charlson Comorbidity Index (CCI)* ≥ 3 points	11 (17.2)	3 (13.0)	0.64
*Menopausal status*			0.14
Premenopausal	17 (26.6)	10 (43.5)	
Perimenopausal	1 (1.6)	1 (4.3)	
Postmenopausal	43 (67.2)	11 (47.8)	
Unknown	3 (4.7)	0 (0)	
Male patient	0 (0)	1 (4.3)	
* HR*** + ***(missing n* = *1)*	49 (76.6)	17 (73.9)	0.80
* HER2*** + ***(missing n* = *2)*	16 (25.0)	5 (21.7)	0.75
*Type of BC therapy*^b^			
Chemotherapy	28 (43.8)	14 (60.9)	0.16
Endocrine therapy	39 (60.9)	12 (52.2)	0.46
Targeted therapy	23 (35.9)	9 (39.1)	0.79
Osteoprotective therapy	35 (54.7)	12 (52.2)	0.84
Immunotherapy	2 (3.1)	1 (4.3)	0.78
*Time since first diagnosis of metastasis*			0.24
0 years	37 (57.8)	10 (43.5)	
≥ 1 year	27 (42.2)	13 (56.5)	
*Number of concurrent metastases*			0.81
1 metastasis	38 (59.4)	13 (56.5)	
≥ 2 metastases	26 (40.6)	10 (43.5)	
* Number of concomitant drugs* ≥ *5 (polypharmacy)*	51 (79.7)	18 (78.3)	0.89
* Perceived health status – EORTC-QL-2* < *4 (missing n* = *3)*	13 (20.3)	7 (30.4)	0.29
* Quality of life (QoL) – EORTC-QL-2* < *4 (missing n* = *3)*	14 (21.9)	4 (17.4)	0.69
* At least moderate depressive symptoms – PHQ-2 summary score* ≥ *3* *(missing n* = *1)*	18 (28.1)	4 (17.4)	0.31
* At least moderate anxiety symptoms – GAD-7 summary score* ≥ *3 (missing n* = *1)*	10 (15.6)	5 (21.7)	0.51
* Worse physical functioning – PROMIS-PF-4a T-score* ≥ *47 (missing n* = *2)*	27 (42.2)	13 (56.5)	0.26
* Experienced pain level* ≥ *2 (missing n* = *2)*	38 (59.4)	16 (69.6)	0.43
* Better perceived physician’s empathy – CARE scale* ≥ *14 (missing n* = *11)*	34 (53.1)	9 (39.1)	0.32
* Feeling slight or not informed in at least one aspect of BC therapy* *(missing n* = *6)*	28 (43.8)	13 (56.5)	0.39
* Moderate to high support needs in at least one health care area (missing n* = *5)*	20 (31.3)	13 (56.5)	0.08
*At least slight impairment due to side effects*^c^			
Infections	15 (23.4)	8 (34.8)	0.36
Metabolic and nutritional disorders	17 (26.6)	16 (69.6)	< 0.001
Cardiovascular side effects	12 (18.8)	7 (30.4)	0.43
Side effects of the kidney or urinary tract	27 (42.2)	14 (60.9)	0.17
Side effects of the respiratory system	15 (23.4)	11 (47.8)	0.08
Side effects of the eyes	19 (29.7)	12 (52.2)	0.09
Side effects of the nervous system	23 (35.9)	15 (65.2)	0.04
Side effects of the gastro-intestinal system	30 (46.9)	15 (65.2)	0.31
Side effects of the skin/hair loss	37 (57.8)	15 (65.2)	0.82
Side effects of the musculo-skeletal system	36 (56.3)	17 (73.9)	0.33

^a^Total number of patients included in the subgroup analysis *n* = 87 (*n* = 6 patients with missing information on adherence at baseline)

^b^More than one therapy type per patient possible

^c^More than one side effect per patient possible

## Discussion

The aim of the present study was to determine adherence and to identify potential factors influencing adherence in patients with advanced or mBC using self-report and Bavarian cancer registry data. We found self-report adherence rates in term of persistence of 75.3% and in terms of quality of execution of 86.0%, respectively, aligning with the findings of other studies on mBC [1, 38, 39]. The adherence in our study is higher than that described in most reviews on early BC [14–31, 33]. Of note, the present study did not merely inquire about adherence to BC therapy; it also included adherence to different types of therapies. As supportive therapy and medication for comorbidities were most affected by non-adherence, adherence to BC therapy may, in fact, be higher in our cohort. However, it was hypothesised that adherence levels in the overall mBC population might be lower, as patients participating in studies are usually more engaged in their therapies [1].

The most frequent reasons for non-adherence encompassed medications beliefs (e.g. patients stated that they do not need a certain medication), forgetfulness, and side effects. In addition, patients impaired by metabolic and neurological side effects were more prone to be non-adherent. Similar, daCosta DiBonaventura et al. (2014) found that forgetfulness and side effects were among the most common reasons for non-adherence [1]. However, they did not include a question on medication beliefs. We further identified, that patients living in a partnership and receiving support from a relative were more likely to be non-adherent. Furthermore, as described in several systematic reviews on early BC, patients with reduced satisfaction with physician’s empathy had poorer adherence [19, 23, 24, 27, 28]. Consistent with findings reported by Figueiredo Junior and Forones (2014), no such association was found for PROs [38]. Furthermore, no association was found for other known factors, such as comorbidities, and HR/HER2 status [31].

Contrary to current evidence, younger advanced or mBC patients (18–49 years) in the present study were more likely to be non-adherent [31]. In general, our study cohort was younger compared to most mBC patients in literature [50, 51]. In younger patients, advanced or mBC is often more aggressive [52, 53]. Consequently, these patients may experience a greater degree of impairment, a higher need for support, more aggressive treatments, and more side effects. These findings indicate that less complex treatment regimens and less aggressive treatments might be linked to improved adherence [31]. Furthermore, patients might benefit from better education on potential side effects and the use of reminders [20, 54].

In the present cohort, supportive therapy and medication for comorbidities were most affected by non-adherence. This indicates that patients may particularly benefit from tailored strategies promoting adherence to non-BC therapies. Indeed, treatment for comorbidities was shown to be associated with non-adherence [7, 55]. Further research is needed to investigate the potential of different interventions to promote adherence to different therapy types in patients with advanced or mBC.

In the cancer registry data, the persistence was 78.3%. It should be noted that the observed five-year treatment pause in three patients was probably due to a lack of reporting of treatments to the cancer registry. Evidence on persistence in advanced or mBC patients is scarce. To the best of our knowledge, no other studies have used cancer registry data to analyse persistence in (m)BC patients. In studies relying on US claims data, similar persistence rates (about 80.0%) were observed [56, 57]. Particularly for bisphosphonates, lower mean persistence rates were reported in studies using claims data (36.4–92.0%) [58, 59]. Further studies are needed to explore the potential of cancer registry data for persistence analyses and to investigate the reasons of potential therapy pauses or discontinuation in advanced or mBC.

### Strengths and limitations

To the best of our knowledge, this was the first study evaluating adherence in a German cohort of advanced or mBC patients. The main strength of our study is that we combined self-report and Bavarian cancer registry data to assess adherence, as it is recommended to apply more than one method [11]. However, the completeness of the cancer registry data is contingent on what is reported to the cancer registry. We cannot rule out the possibility that the therapy pauses found were due to a lack of reporting. Besides routine data, adherence methods are affected by the Hawthorne effect, which posits that every measurement might also enhance patients’ adherence [11]. Furthermore, a major limitation of self-report adherence measurement is its susceptibility to manipulation and reporting bias due to social desirability and recall bias. Given the high self-reported adherence rates in the present cohort, reporting bias in particular should be considered. Future studies may consider applying self-report measures together with objective measures such as electronic monitoring, to enhance the reliability of the results.

A further limitation is that adherence could not be analysed stratified by specific drugs as in clinical practice, adherence may vary considerably depending on the pharmacologic agent for example due to different side effects [14, 19, 21, 25, 27, 30, 60]. However, regarding the type of BC therapy, no statistically significant differences were observed. Moreover, we focused on adherence to oral treatments and did not assess non-adherence in terms of missed appointments e.g. for scheduled intravenous treatments. Failure to attend such sessions is a clinically relevant indicator of non-adherence [61]. Future studies should incorporate questions on adherence to these appointments and should differentiate between oral versus intravenous treatments. Additionally, complexity of dosing regimens and scheduling burden may be important to be considered.

As previously described, our advanced or mBC cohort is comparable to other (advanced/m)BC patients in terms of expression of HR and HER2 status, and metastatic sites [48, 62–66]. However, a major limitation results from the small sample size. Thus, no time trends in medication adherence could be identified, factors influencing medication adherence could not be evaluated in multivariate analysis, and the generalisability of the results may be limited. Last, our results are limited by the heterogeneous nature of the data collection. Only patients recruited before August 2023 were followed for 12 months, and up to 44% of these patients were lost to follow-up, further reducing the sample size in the follow-up. Therefore, the possibility of attrition bias cannot be discounted limiting the generalisability of our findings. Potential reasons described in literature for this attrition in the present patient collective may be the patients’ age [67], the burden of their advanced or mBC [68], and their forgetfulness [69]. Methods undertaken in the follow-up process to enhance the patients’ retention were up to three postal reminders and up to three telephone contacts. The approach to the data collection was necessitated by measures to increase the sample size for the primary aim of the BRE-BY-MED study.

## Conclusion

The observed adherence rates to drug therapy in patients with advanced or mBC using self-report and Bavarian cancer registry data suggested that further research is needed to investigate the potential of tailored interventions to improve adherence to supportive therapy and medication for comorbidities in patients with advanced or mBC considering the reasons for non-adherence. In addition, further research is needed to investigate the reasons of therapy pauses or discontinuation in advanced or mBC considering the the potential of cancer registry data for persistence analyses.

## Data Availability

Data are available on request to the authors.

## Abbreviations

BC: Breast cancer
advanced or mBC: Advanced or metastatic breast cancer
CCI: Charlson Comorbidity Index
eCR: Electronic case report form
HER2: Human epidermal growth factor receptor 2
HR: Hormonal receptor
PRO: Patient reported outcome
PROMIS-PF-4a: PROMIS® v2.0 Physical Function SF 4a
QoL: Quality of life
